# Supplementary material for: A Survey of Mobile Apps for the Care Management of Patients with Dementia
Source: Healthcare (Basel). 2022 Jun 23;10(7):1173. doi: 10.3390/healthcare10071173 (PMC9317040; doi:10.3390/healthcare10071173)
Supplement: Supplementary file 1 [file healthcare-10-01173-s001.zip › healthcare-1748408-supplementary.pdf]

Supplement Table S1. Summary of included APP characteristics.

Code	APP Name	Country	Developer	Affiliations	Website	Last Update	Download
87	Accessible Alzheimer's and Dementia Care	US	AHCGLOBAL, INC.	Commercial	https://apps.apple.com/us/app/accessibl e-alzheimers-and-dementia-care/id1193296364	NA	500+
88	Alzheimer's and Dementia Care	US	Accessible home health care	Commercial	https://play.google.com/store/apps/detai ls?id=tritantra.ahc2	NA	500+
89	Alzheimer's Daily Companion	US	Home Instead Senior Care	Commercial	https://apps.apple.com/us/app/alzheime rs-daily-companion/id696976537	NA	5000+
90	Alzheimer's Manager	US	Point of Care LLC	Commercial	https://apps.apple.com/tw/app/alzheime rs-manager/id1364064302	20220302	0
91	Cogni Care	UK	CongniHealth Ltd	Commercial University	https://apps.apple.com/us/app/cogni/id9 02479424	20210107	5000+
92	Dementia Advisor	Canada	Sinai Health System	Government Health System	https://apps.apple.com/us/app/dementia -advisor/id1156249743	20190920	1000+
93	Dementia Caregivcer Solutions	Canada	Lorenzo Gentile	Commercial	https://apps.apple.com/tw/app/dementi a-caregiver-solutions/id969874075?l=zh	20150710	0
94	Dementia Stages Ability Model	US	Positive Approach, LLC	Commercial	https://apps.apple.com/us/app/dementia -stages-ability-model/id1528128949	NA	1000+
95	DementiAssist	US	Baylor Scott and White Health	University	https://apptopia.com/ios/app/92651612 2/about	20190518	5000+
96	DemKonnect	UK	Nightingales Medical Trust	NGO	https://play.google.com/store/apps/detai ls?id=mobi.appyapp.demkonnect	20220609	500+
97	Inspo-Alzheimer'sCare giving	US	Inspo Labs	NA	https://apps.apple.com/hk/app/inspo- alzheimers-caregiving/id1545633090	20220226	0
98	Remember Me-Caregiver	US	Daniel Leal	NA	https://apps.apple.com/tw/app/rememb er-me-caregiver/id1481000591	20191005	0
99	Care4Dementia	Australia	Univ of New South Wales	University	https://apps.apple.com/tw/app/care4de mentia/id1029281368	NA	0
